# Cholesterol lowering drug use and breast cancer survival: the Multiethnic Cohort Study

**DOI:** 10.1007/s10549-021-06360-y

**Published:** 2021-08-30

**Authors:** Nafeesa Moksud, Lenora W. M. Loo, Juan Yang, Chiung-Yu Huang, Christopher A. Haiman, Loïc Le Marchand, Lynne R. Wilkens, Iona Cheng

**Affiliations:** 1 Department of Epidemiology and Biostatistics, University of California, San Francisco, 550 16th Street, San Francisco, CA 94158 USA; 2 Cancer Biology Program, University of Hawaii Cancer Center, Honolulu, HI USA; 3 Population Sciences in the Pacific Program, University of Hawaii Cancer Center, Honolulu, HI USA; 4 Department of Preventive Medicine, Keck School of Medicine, University of Southern California, Los Angeles, CA USA

**Keywords:** Breast cancer, Survival, Mortality, Cholesterol lowering drug, Multiethnic Cohort

## Abstract

**Purpose:**

Prior studies conducted primarily in white populations have suggested that pre-diagnostic cholesterol lowering drugs (CLDs) improved survival among women with breast cancer (BC). However, this association had not been well characterized in diverse racial/ethnic populations. We investigated whether pre-diagnostic CLD use is associated with all-cause and BC-specific mortality among female BC cases of the Multiethnic Cohort (MEC).

**Methods:**

CLD use was ascertained through questionnaires administered in 2003–2008. A total of 1448 incident BC cases were identified by linkage to SEER cancer registries in Hawaii and California from 2003 to 2014. Multivariable Cox regression was conducted to estimate hazard ratios (HR) and 95% confidence intervals (CI) of the associations of pre-diagnostic CLD use with all-cause and BC-specific mortality, adjusting for tumor characteristics, first course of treatment, health behaviors, co-morbidities, and demographics. Subgroup analyses by stage and hormone receptor status were conducted for all-cause mortality.

**Results:**

There were 224 all-cause and 87 BC-specific deaths among the 1448 BC cases during a median follow-up of 4.5 years after diagnosis. Women with BC who ever used CLDs had a 27% lower hazard of all-cause mortality (HR 0.73, 95% CI 0.54–0.98) and 17% lower hazard of BC-specific mortality (HR 0.83, 95% CI 0.49–1.39) compared to never users. CLD use reduced mortality among women with advanced-stage tumors and hormone receptor-positive breast tumors (HR 0.54 95% CI 0.33–0.90; HR 0.69, 95% CI 0.48–0.99, respectively).

**Conclusion:**

These findings demonstrate an improved survival associated with CLD use prior to diagnosis in a multiethnic population of women with BC.

**Supplementary Information:**

The online version contains supplementary material available at 10.1007/s10549-021-06360-y.

## Introduction

Breast cancer is the most commonly diagnosed cancer among women in the United States, and advances in disease management and the promotion of breast cancer screening have improved the survival rates of breast cancer. Recent endeavors in re-purposing old drugs for novel uses [1–4] sparked interest in investigating the effects of commonly used medications on health outcomes in cancer survivors [5–7]. Cholesterol lowering drugs are a good candidate as they are commonly prescribed amongst the general population. Additionally, some cholesterol lowering drugs such as statins (hydroxymethylglutaryl-CoA (HMG-CoA) reductase inhibitors) were discovered to have oncotherapeutic potential in breast cancer cell lines [8]. While the various purported mechanisms of how statins have anti-tumor properties are reported extensively elsewhere [9, 10], the most commonly reported mechanism is that statins inhibit the rate limiting step in the mevalonate pathway, preventing the synthesis of essential metabolites for multiple cellular processes, including cellular proliferation, migration, and survival [11–14].

Several epidemiologic cohort studies have shown that use of cholesterol lowering drugs confers benefit on all-cause and breast cancer-specific mortality among breast cancer cases [15–20]. In the largest cohort study to date, conducted among 20,559 Swedish women with breast cancer, Borgquist et al. reported all-cause mortality hazard ratios of 0.76 (95% CI 0.66–0.88) and 0.89 (95% CI 0.82–0.96) for pre-and post-diagnostic statin use, respectively, compared to never users. The HRs for breast cancer-specific mortality was 0.77 (95% CI 0.63–0.95) and 0.83 (95% CI 0.75–0.93) for pre- and post-diagnostic statin use, respectively, compared to never users [19]. The majority of studies on the association between statins and breast cancer outcomes were conducted among cohorts of Scandinavian and Western European women [15–19, 21, 22].

Two U.S. studies [23–25] with substantial non-white breast cancer cases did not report race/ethnicity-specific findings. Li et al. [25] reported findings on a cohort of 1523 women with breast cancer (60% white, 31% African American, 8% other breast cancer cases) with all-cause mortality hazards of 0.38 (95% CI 0.17–0.85) for women who had used statins for 5 years or longer. Shaitelman et al. [24] reported all-cause mortality hazards of 0.74 (95% CI 0.20–2.77) and breast cancer-specific mortality hazards of 0.70 (95% CI 0.47–1.03) in a cohort of 869 women with triple negative breast cancer (60% white, 22% African American, 14% Hispanic, 5% other). Another study by Leiter et al. [23] examined racial differences in breast cancer prognosis using the Nottingham Prognostic Index in 100 African American and 487 white breast cancer cases. Leiter et al. reported that pre-diagnostic statin use could not explain the better prognosis for white breast cancer cases in comparison to African American cases [23]. As there have been limited studies conducted among diverse racial/ethnic groups, the objective of this report was to examine the association between use of cholesterol lowering drugs prior to diagnosis and mortality in a multiethnic population of breast cancer cases.

## Methods

The Multiethnic Cohort (MEC) Study was established to investigate diet and lifestyle factors and cancer in five racial/ethnic groups—African American, Japanese Americans, Latino American, Native Hawaiian and white [26]. Participants were recruited from a population-based sampling frame, mainly using driver’s license records from Hawaii and Southern California. Participants were enrolled into the cohort in 1993 through 1996 by completing a baseline self-administered questionnaire that surveyed demographics, body size, personal and family medical history, occupational history, food intake, physical activity, and medication use. This questionnaire with minor modifications was repeated in 2003–2008 (Qx3). Further details on the design, implementation, and composition of the MEC study are described elsewhere [26].

Use of cholesterol lowering drug use was not included at baseline, as use was not common at that time, but was added at follow-up questionnaire (QX3). The following question at QX3 was asked to assess cholesterol lowering drug use: *Have you ever taken any of the following medications at least two times per week (for 1 month or longer)*? The question was posed for high cholesterol medication, with examples of brand names provided (Lipitor, Mevacor, Zocor, Pravachol, Lopid or other). Participants responded “no,” “yes, but not at this time,” or “yes, currently.” If participants answered “yes,” they were asked to mark the length of medication use as 1 year or less, 2–3 years, 4–5 years, 6–10 years, or 11 years or more.

Cancer cases in the MEC were identified via linkages to Hawaii and California cancer registries both members of National Cancer Institute Surveillance, Epidemiology, and End Results Program (SEER) Program. Information on date of diagnosis, tumor characteristics (SEER summary stage of disease, hormone receptor status, lymph node involvement), and first course of treatment (surgery, hormone therapy) were obtained from the registries. Vital status and cause of death were collected through state death certificates and linkage with the National Death Index. For this study, eligible MEC women were considered only if they had completed QX3 (sent and collected from 2003 to 2008), had no breast cancer diagnosis prior to QX3, and had an invasive breast cancer diagnosis after receipt of QX3 through 2014. We excluded breast cancer cases, who did not provide data on cholesterol lowering medication use (*n* = 121), premenopausal women (*n* = 7) or women with unknown menopausal status (*n* = 4), resulting in a final study population of 1448 breast cancer cases with cholesterol lowering drug use ascertained prior to diagnosis. These women were followed for vital status from date of diagnosis (2003–2014) to study end date (31st December 2014), with a median follow-up time of 4.5 years (SD ± 2.86 years).

### Statistical analysis

We used chi-square and Student’s *t*-tests to evaluate differences in study characteristics by pre-diagnostic cholesterol-lowering drug use. Multivariable-adjusted hazard ratios (HRs) and 95% confidence intervals (CI) for all-cause mortality were estimated using Cox proportional hazards regression, using age as the time metric. Follow-up started at the age of breast cancer diagnosis and ended at the age of death or age at end of follow-up (12/31/2014). Models were adjusted for race/ethnicity (African American, Japanese Americans, Latino American, Native Hawaiian, and white), level of education (high school graduate or less, college or vocational school, graduate or professional school), and the following covariates collected at QX3: body mass index (normal, underweight, overweight, obese class I, obese class II/III, unknown), daily energy intake (< 1164 cal, 1164–1550 cal, 1550–2037 cal, > 2037 cal), Alternate Healthy Eating Index (AHEI) Score [27] (27–60, 60–67, 67–74, 74–101), age at menarche (< 11 years, 11–12 years, 13–14 years, 15–16 years, ≥ 17 years, unknown), cardiovascular disease risk (none, hypertensive or taking hypertension medications, history of cardiovascular disease or stroke), diabetes (not diabetic, diabetic), tumor stage at diagnosis (localized, regional, distant, unknown), lymph node status at diagnosis (lymph node negative, 1 or more lymph node positive, unknown), estrogen and progesterone receptor (ER and PR) status (ER+PR+, ER+PR−, ER−PR+, ER−PR−, unknown), surgery (no, yes), and hormone therapy (no, yes). Chemotherapy and mammography screening were found not to be associated with mortality among this sample and were not included as covariates. Cholesterol lowering drug use was categorized as “never”, “past”, and “current”. “Past” and “current” users were also combined to make an “ever” user category. Duration of cholesterol lowering drug use was categorized into “less than or equal to 3 years of use” and “more than 3 years of use”. Subgroup analysis was conducted by race/ethnicity, tumor stage (localized; advanced: regional and distant), and hormone receptor status (hormone receptor positive: ER+ and/or PR+; hormone receptor negative: ER− and PR−). We assessed heterogeneity of associations for cholesterol lowering drug use and all-cause mortality by race/ethnicity, stage, and hormone receptor status using a global test of interaction. Multivariable adjusted hazard ratios were also calculated for breast-cancer specific mortality. The proportional hazards assumption was assessed by examining log(− log(survival function)) plots for diverging or crossing survival curves over time and by assessing the Schoenfeld residuals. To explore racial/ethnic differences among breast cancer cases, we evaluated cholesterol lowering drug use prevalence, demographics, lifestyle factors, comorbidities, and treatment factors across the five racial/ethnic groups. In addition, we applied the Inverse Probability of Treatment Weighting [28] approach to all-cause and breast-cancer specific models of ever versus never cholesterol lowering drug use. The weight was determined for each individual as *w* = *Z*/*e* + ((1 − *Z*))/(1 − *e*), where *Z* is the indicator variable denoting whether CLD was used or not; and e denoting the predicated probability of cholesterol lowering drug use (ever versus never) conditioned on all other covariates adjusted for in the original multivariable Cox model. SAS version 9.4 (SAS Institute Inc., Cary, NC) and Stata 16.1 (College Station, TX: StataCorp LLC.) were used for analyses with a two-sided *p*-value of < 0.05 considered to be statistically significant.

## Results

Our final study population consisted of 1448 breast cancer cases (54.8% never users, 6.7% past users, and 38.5% current users of cholesterol lowering drugs). During the median follow-up time of 4.5 years after diagnosis, 224 cases died, of which 87 deaths were due to breast cancer. The median follow-up time did not differ significantly by cholesterol lowering drug use (4.52 years for ever users, 4.47 years for never users). We observed racial/ethnic differences between use of cholesterol lowering drugs among our breast cancer cases (Table 1). Among ever users, the highest percentage of use was seen among Japanese American cases (55.9%) followed by Native Hawaiian (52.3%), Latino American (44.7%), African American (39.4%), and white (36.4%) cases. Cholesterol lowering drug use was more likely in older or diabetic cases, those with higher BMIs and higher cardiovascular risk, and those receiving hormonal therapy (Table 1).

**Table 1:** Study characteristics of female breast cancer cases in the MEC diagnosed between 2003 and 2014 by cholesterol lowering drug use

	Never user (*n* = 793)		Ever user (*n* = 655)		*p* value
Demographics^a^					
Age at diagnosis, mean ± SD, year	72.9	± 8.24	74.5	± 7.95	** ≤ 0.001**
Years of follow-up, mean ± SD, year	4.47	± 2.82	4.52	± 2.90	0.73
Race/Ethnicity, *n*, %					**0.001**
African American	120	60.6%	78	39.4%	
Japanese American	224	44.1%	246	55.9%	
Latino American	115	55.3%	93	44.7%	
Native Hawaiian	67	47.7%	85	52.3%	
White	267	63.6%	153	36.4%	
*Marital status, *n*, %					0.13
Married	418	53.7%	360	46.3%	
Separated/Divorced	140	59.3%	96	40.7%	
Widowed	161	52.4%	146	47.6%	
Never married	63	63.0%	37	37.0%	
*Level of education, *n*, %					**0.03**
High school graduate or less	249	50.1%	248	49.9%	
College or vocational school	390	56.4%	301	43.6%	
Graduate/professional school	146	59.1%	101	40.9%	
Health behaviors and past medical history^1^					
Body Mass Index, kg/m^2^, mean + SD	26.7	± 6.01	28.3	± 6.26	** ≤ 0.001**
Daily caloric intake, kcal, mean + SD	1682	± 718	1679	± 784	0.96
Alternate Healthy Eating Index Score, mean + SD	67.0	± 10.20	66.7	± 8.96	0.51
*Age at menarche, *n*, %					0.12
< 11 years	58	45.3%	70	54.7%	
11–12 years	346	53.9%	296	46.1%	
13–14 years	304	58.2%	218	41.8%	
15–16 years	73	55.3%	59	44.7%	
> 17 years	5	55.6%	4	44.4%	
Cardiovascular disease, *n*, %					** ≤ 0.001**
None	319	73.5%	115	26.5%	
Hypertensive, or taking hypertension medications	409	49.3%	421	50.7%	
History of cardiovascular disease or stroke	65	35.3%	119	64.7%	
Diabetes, *n*, %					** ≤ 0.001**
Not diabetic	718	60.3%	473	39.7%	
Diabetic	75	29.2%	182	70.8%	
Tumor characteristics^b^					
*Stage at diagnosis, *n*, %					0.52
Localized	578	54.2%	488	45.8%	
Regional	181	56.4%	140	43.6%	
Distant	26	61.9%	16	38.1%	
*Lymph node status at diagnosis, *n*, %					0.37
All nodes negative	588	53.9%	503	46.1%	
1 or more nodes positive	176	56.8%	134	43.2%	
*Estrogen and progesterone receptor status, *n*, %					0.13
ER+/PR+	544	54.0%	464	46.0%	
ER+/PR−	109	60.2%	72	39.8%	
ER−/PR+	3	27.3%	8	72.7%	
ER−/PR−	114	57.0%	86	43.0%	
*Surgery, *n*, %					0.75
No	37	52.9%	33	47.1%	
Yes	748	54.8%	616	45.2%	
*Hormone therapy, *n*, %					0.03
No	370	57.5%	274	42.5%	
Yes	376	51.6%	352	48.4%	

*p*-values provided by using chi-squared and Student’s *t*-tests. Bolded values indicate *p*-values ≤ 0.05

*SD* standard deviation, *ER* estrogen receptor, *PR* progesterone receptor

*Counts in these categories may not add up to totals due to participants with missing information

^a^Demographics, health behavior and past medical history, and cholesterol lowering drug use was collected from MEC Qx3 administered between 2003 and 2008

^b^Tumor data was collected at time of diagnosis (2003–2014)

Compared to never-users, breast cancer cases who reported having ever used cholesterol lowering drugs prior to diagnosis had a 27% lowered hazard of all-cause mortality (HR 0.73, 95% CI 0.54–0.98, Table 2). A similar pattern of association was observed across race/ethnicity with the exception of white women with breast cancer (Supplementary Table 1; *p* for heterogeneity by race/ethnicity = 0.11).

**Table 2:** Association between cholesterol lowering drug use and all-cause mortality among women diagnosed with breast cancer in the MEC (2003–2014)

Cholesterol lowering drug use	*n* Cases	*n* Deaths	HR	95% CI	*p* value
Never/Ever					
Never (reference)	793	123	1.00		
Ever	655	101	**0.73**	**0.54–0.98**	**0.04**
Never/current/past					
Never (reference)	793	123	1.00		
Past	97	19	0.75	0.43–1.28	0.29
Current	558	82	**0.72**	**0.53–0.99**	**0.05**
Duration of use					
Never (reference)	793	123	1.00		
3 years of use or less	320	50	**0.62**	**0.42–0.91**	**0.01**
More than 3 years of use	309	49	0.90	0.62–1.29	0.57

Adjusted for age at breast cancer diagnosis, race/ethnicity, level of education, body mass index, daily caloric intake, Alternate Healthy Index Score, age at menarche, cardiovascular disease, diabetes, tumor stage, lymph node status, hormone receptor status, surgery, and hormone therapy

Bolded values indicate *p*-values ≤ 0.05

Lower mortality was seen for current users (HR 0.72, 95% CI 0.53–0.99) and suggested for past users (HR 0.75, 95% CI 0.43–1.28) in comparison to never-users (Table 2). There was no significant difference in the association between past users and current users (*p* = 0.34). In addition, cholesterol-lowering drug use for less than or equal to 3 years was associated with lower all-cause mortality in comparison to never use (HR 0.62, 95% CI 0.42–0.91, Table 2). No significant difference in association was found between breast cancer cases that used cholesterol lowering drugs for 3 years or less versus more than 3 years (*p* = 0.34). For breast cancer-specific mortality, breast cancer cases who reported having ever versus never used cholesterol lowering drugs had a 17% lowered hazard of mortality (HR 0.83, 95% CI 0.49–1.39, Supplementary Table 2) that did not reach statistical significance.

Table 3 presents the association between pre-diagnostic cholesterol lowering drug use and all-cause mortality among breast cancer cases by stage of disease. For localized breast cancer, ever use of cholesterol lowering drugs had a 9% lower hazard for all-cause mortality (HR 0.91, 95% CI 0.59–1.38) compared to never users. For regional/distant breast cancer, there was a 46% reduction in the hazard for all-cause mortality (HR 0.54 95% CI 0.33–0.90). However, there was no significant difference in the hazard ratios by stage (*p* for heterogeneity by stage = 0.48).

**Table 3:** Association between cholesterol lowering drug use and all-cause mortality among women diagnosed with breast cancer in the MEC (2003–2014) by tumor stage

	*n* Cases	*n* Deaths	HR	95% CI	*p* value
Localized					
Never use (reference)	578	56	1.00		
Ever use	488	60	0.91	0.59–1.38	0.65
Advanced^a^					
Never use (reference)	207	65	1.00		
Ever use	156	36	**0.54**	**0.33–0.90**	**0.02**

Adjusted for age at breast cancer diagnosis, race/ethnicity, education, body mass index, daily caloric intake, Alternate Healthy Index Score, age at menarche, cardiovascular disease, diabetes, lymph node status, hormone receptor status, surgery, and hormone therapy. *p*-heterogeneity by stage = 0.48

Bolded values indicate *p*-values ≤ 0.05

^a^Advanced stage includes regional- and distant-stage tumors

For hormone receptor-positive breast cancer, ever use of cholesterol lowering drugs prior to diagnosis had a 31% lower hazard for all-cause mortality (HR 0.69, 95% CI 0.48–0.99) compared to never user (Table 4). For hormone negative breast cancer, no association between cholesterol lowering drug use and mortality was observed. However, no evidence of differences between the hazard ratios was found (*p* for heterogeneity by hormone receptor status = 0.51).

**Table 4:** Association between cholesterol lowering drug use and all-cause mortality among women diagnosed with breast cancer in the MEC (2003–2014) by hormone receptor status

	*n* Cases	*n* Deaths	HR	95% CI	*p* value
Hormone receptor (+)^a^					
Never use (reference)	656	83	1.00		
Ever use	544	71	**0.69**	**0.48–0.99**	**0.05**
Hormone receptor (−)^a^					
Never use (reference)	114	32	1.00		
Ever use	86	23	1.04	0.52–2.07	0.92

Adjusted for, age at breast cancer diagnosis, race/ethnicity, education, body mass index, daily caloric intake, Alternate Healthy Index Score, age at menarche, cardiovascular disease, diabetes, tumor stage, lymph node status, surgery, and hormone therapy. *p*-heterogeneity by hormone receptor status = 0.51

Bolded values indicate *p*-values ≤ 0.05

^a^Hormone receptor (+) includes tumors that are either estrogen receptor-positive or progesterone receptor-positive. Hormone receptor (−) includes tumors that are estrogen receptor and progesterone receptor-negative

Supplementary Table 3 describes study characteristics of cholesterol lowering drug use across each racial/ethnic group. Native Hawaiian breast cancer cases (56%) had the highest prevalence of ever use of cholesterol lowering drugs followed by Latino American (52%), Japanese American (45%), African American (39%), and white (36%) breast cancer cases. Age at breast cancer diagnosis was similar across racial/ethnic groups with the exception of a younger mean age of diagnosis among Native Hawaiians (70.8 years). Japanese American breast cancer cases had lower BMIs compared to the other racial/ethnic groups (56% had normal BMI). While cardiovascular disease was similar across race/ethnicity, Latino American and Native Hawaiian breast cancer cases had a higher proportion of diabetes (29% and 24%, respectively). Across all racial/ethnic groups most women were likely to be diagnosed with localized breast cancer. African American breast cancer cases had a higher proportion of hormone receptor negative breast cancer compared to other racial/ethnic groups (22%).

Supplementary Table 4 presents the association between pre-diagnostic cholesterol lowering drug use and all-cause and breast cancer-specific mortality using the Inverse Probability of Treatment Weighting approach [28]. For all-cause mortality, ever use of cholesterol lowering drugs showed a 20% lowered hazard (HR 0.80, 95% CI 0.67–0.95) compared to never use. For breast cancer-specific mortality, a lowered hazard was observed for ever versus never use (HR 0.88, 95% CI 0.66–1.18) that was not statistically significant.

## Discussion

In this multiethnic population, all-cause mortality was lower among women with breast cancer who reported having ever used cholesterol lowering drugs before diagnosis. Compared to never users, current users of cholesterol lowering drugs at assessment also experienced a significantly reduced all-cause mortality compared to never users, as did those who had used cholesterol lowering drugs for 3 years or less. Breast cancer specific-mortality was also suggested to be associated with pre-diagnostic cholesterol-lowering drug use. Women with advanced stage tumors had a lower all-cause mortality associated with ever use of cholesterol lowering drug use, compared to never users. In addition, women with hormone receptor-positive breast tumors had lower all-cause mortality associated with ever use of cholesterol lowering drug use.

While our questionnaire did not differentiate between the classes of cholesterol lowering drugs, we believe most of our cholesterol lowering drug users were taking statins, as statins have been the cornerstone of management for high blood cholesterol [29] since it was first approved by the Food and Drug Administration in 1987. Trends of statin therapy among adults since then have increased during the study period [30].

Our results of the associations of pre-diagnostic cholesterol lowering drug use with all-cause and, suggestively breast cancer-specific mortality agree with results from previous studies and meta-analyses [20, 31]. A comprehensive meta-analysis by Wu et al. [20] reported a pooled result from five studies on statin use and breast cancer survival, showing a beneficial effect of pre-diagnostic statin use on overall survival (HR 0.68, 95% CI 0.54–0.84) and on breast cancer-specific mortality (HR 0.72 95% CI 0.53–0.99). A later meta-analysis by Liu et al. [31] also reported similar estimates on all-cause mortality (HR 0.72, 95% CI 0.58–0.89) and breast cancer specific-mortality (HR 0.73, 95% CI 0.59–0.92) based on seven studies.

Liu et al. [31] additionally performed a subgroup meta-analysis by follow-up time as a measure to assess duration of statin use. They found that longer follow-up time did not show a significant association between statin use and all-cause mortality (HR 0.95, 95% CI 0.75–1.19); yet less than 4 years of follow-up showed a protective effect of statins against all-cause mortality (HR 0.61, 95% CI 0.45–0.80). A more recent cohort study by Li et al. [25] found that statin use more than 5 years was associated with improved overall survival (HR 0.38, 95% CI 0.17–0.85) compared to use less than 3 years (HR 1.50, 95% CI 0.79–2.86) and between 3 and 5 years (HR 1.07, 95% CI 0.52–2.15). In our study, cholesterol-lowering drug use for 3 years or less was associated with lower all-cause mortality in comparison to never use. Due to limited sample size, we were unable to evaluate a larger range in the duration of cholesterol-lowering drug use or to evaluate breast cancer-specific mortality. A possible reason for the differences in hazard ratios by length of use in our cohort of cases may be due to the progression of cardiovascular symptoms over time, increasing the risk for all-cause mortality among cases with longer duration of use.

Murtola et al. [22] reported a HR of 0.58 (95% CI 0.49–0.70) among current users of statins associated with localized breast cancer versus statin never users, while current users among women with metastatic breast cancer had a HR of 0.66 (95% CI 0.47–0.92) versus statin never users. Our study found cholesterol lowering drug use reduced all-cause mortality among women with advanced stage (regional and distant) tumors compared to never users. No statistically significant reduction in mortality was seen among cholesterol lowering drug users with localized tumors in our cohort.

Differences in the associations between cholesterol lowering drug use and mortality among breast cancer cases by hormone receptor status has been investigated in some studies [16, 18, 24]. McMenamin et al. [16] found borderline statistically significant evidence of reduced overall mortality in a subgroup analysis of 1495 women with ER-positive breast tumors, who had ever used statins (HR 0.85, 95% CI 0.72, 1.01). Our findings for hormone receptor-positive breast cancers suggest beneficial effects on overall survival from cholesterol lowering drug use as reported in other studies.

A possible biological mechanism by which cholesterol lowering drug use influences overall mortality may be due to the inhibition of the mevalonate pathway to reduce cholesterol synthesis that is relevant for multiple pathways for cholesterol-related diseases. Specifically for the reduced risk for hormone receptor-positive breast cancer mortality, cholesterol is the building block for hormones (e.g. estrogen and progesterone) and the recently identified selective endogenous estrogen receptor modulator (SERM), 27-hydroxycholesterol; therefore, inhibition of cholesterol synthesis may result in reduced cellular proliferation for hormone receptor-positive breast tumors [32].

A study by the Breast International Group based on clinical trial data of 7963 postmenopausal women with early stage hormone receptor-positive invasive breast cancer showed that initiation of cholesterol-lowering medications during any endocrine therapy was associated with improved disease-free survival compared to never users (HR 0.79, 95% CI 0.66–0.95) [33]. Shaitelman et al. [24] conducted a study of statin use among triple-negative (ER-negative, PR-negative, HER2-negative) breast cancer and found no significant association between statin use and overall survival (HR 0.74, 95% CI 0.20–2.77).

Reports on the use of cholesterol lowering drug across race/ethnicity for breast cancer survivors are sparse. Similar to Leiter et al. [23], our study found similar prevalence of cholesterol lowering drug use between African American and white breast cancer cases. Li et al. [25] and Shaitelman et al. [24] also reported similar prevalence of use among African American cases as our study (39% ever users in all three studies). Our study is the first to report a high prevalence of cholesterol lower drug use among Japanese American (53% ever users) and Native Hawaiian (56% ever users) breast cancer cases compared to White cases (37% ever users).

Our study has limitations to consider. It was based on the assessment of cholesterol lowering drug use at a single time point prior to breast cancer diagnosis with possible misclassification of exposure; yet we would expect this misclassification to be non-differential by vital status. Information on duration and specific types of cholesterol lowering drug use was limited to specific time categories and we were unable to evaluate differences in duration by more refined years due to limited detailed data from our questionnaire. Due to limited sample size and deaths by race/ethnicity, we were unable to examine racial/ethnic-specific associations for breast cancer specific mortality.

As one of the most commonly occurring cancers worldwide, breast cancer is one of the most researched carcinomas. As a result, the survival of women with breast cancer has improved significantly over the past few decades. Yet, continued research is needed to address the persistent disparities in survival, especially disparities that may arise due to modifiable factors associated with race and ethnicity, such as barriers to healthcare, health beliefs and behaviors, and socioeconomic mobility. Established drugs such as cholesterol lowering drugs may be a means to improve survival outcomes, while also being a safer alternative compared to newer drugs with its well-known safety profile. Multiethnic data are a valuable resource to evaluate survival outcomes across racial/ethnic groups.

## Conclusion

In this multiethnic population of breast cancer cases, use of cholesterol lowering drugs reduced all-cause mortality compared to never use. The results from our study are promising as they confirm results found in literature among a multiethnic population. Future studies should examine the effects of post-diagnostic use, time-dependent use and type of cholesterol lowering drug use on breast cancer survival outcomes across racially/ethnically diverse populations.

## Supplementary Information

Below is the link to the electronic supplementary material.

Supplementary file1 (DOCX 28 kb)

## Data Availability

The data that support the findings of this study are available to researchers through the procedures described at https://www.uhcancercenter.org/for-researchers/mec-data-sharing.

## Abbreviations

CLD/CLDs: Cholesterol lowering drug(s)
BC: Breast cancer
MEC: Multiethnic Cohort
HR: Hazard ratio
CI: Confidence interval
HMG Co-A: 3-Hydroxy-3-methyl-glutaryl-coenzyme A reductase
QX3: MEC Questionnaire 3
SEER: Surveillance, Epidemiology, and End Results Program
AHEI: Alternate Healthy Eating Index
ER: Estrogen receptor
PR: Progesterone receptor
HER-2: Human epidermal growth factor receptor 2
