# Supplementary material for: Cholesterol lowering drug use and breast cancer survival: the Multiethnic Cohort Study
Source: Breast Cancer Res Treat. 2021 Aug 30;190(1):165–73. doi: 10.1007/s10549-021-06360-y (PMC8557195; doi:10.1007/s10549-021-06360-y)
Supplement: Supplementary file 1 — Supplementary file1 (DOCX 28 kb) [file 10549_2021_6360_MOESM1_ESM.docx]

| **Supplementary Table 1: Association between cholesterol lowering drug use and all-cause mortality among women diagnosed with breast cancer in the MEC (2003-2014) by race/ethnicity^*^** | | | | | |
| --- | --- | --- | --- | --- | --- |
|  | **n cases** | **n deaths** | **HR** | **95% CI** | **p-value** |
| **African American** | | | | | |
| Never use (Reference) | 120 | 24 | 1.00 |  |  |
| Ever use | 78 | 23 | 0.68 | 0.32-1.47 | 0.33 |
| **Japanese American** | | | | | |
| Never use (Reference) | 224 | 35 | 1.00 |  |  |
| Ever use | 246 | 24 | **0.41** | **0.20-0.84** | **0.01** |
| **Latino American** | | | | | |
| Never use (Reference) | 115 | 20 | 1.00 |  |  |
| Ever use | 93 | 15 | 0.66 | 0.23-1.88 | 0.43 |
| **White** | | | | | |
| Never use (Reference) | 267 | 37 | 1.00 |  |  |
| Ever use | 153 | 25 | 1.30 | 0.69-2.44 | 0.41 |
| *Adjusted for age at breast cancer diagnosis, education, body mass index, daily caloric intake, Alternate Healthy Index Score, age at menarche, cardiovascular disease, diabetes, tumor stage, lymph node status, hormone receptor status, surgery, and hormone therapy. p-heterogeneity by race/ethnicity= 0.11  Effect estimates for Native Hawaiians could not be estimated due to limited sample size.  Bolded values indicate p-values ≤ 0.05 | | | | | |

| **Supplementary Table 2: Association between cholesterol lowering drug use and breast cancer specific-mortality among women diagnosed with breast cancer in the MEC (2003-2014)^*^** | | | | | |
| --- | --- | --- | --- | --- | --- |
| **Cholesterol lowering drug use** | **n cases** | **n deaths** | **HR** | **95% CI** | **p-value** |
| **Never/Ever** | | | | | |
| Never (Reference) | 791 | 52 | 1.00 |  |  |
| Ever | 654 | 35 | 0.83 | 0.49-1.39 | 0.47 |
| *Adjusted for age at breast cancer diagnosis, race/ethnicity, level of education, body mass index, daily caloric intake, Alternate Healthy Index Score, age at menarche, cardiovascular disease, diabetes, tumor stage, lymph node status, hormone receptor status, surgery, and hormone therapy. | | | | | |

| **Supplementary Table 3:**  **Study characteristics of women diagnosed with breast cancer in the MEC (2003-2014) by race/ethnicity** | | | | | | |
| --- | --- | --- | --- | --- | --- | --- |
| **Characteristic** | **African American (n=198)** | **Japanese American (n=470)** | **Latino American (n=208)** | **Native Hawaiian (n=152)** | **White (n=420)** | **Total (n=1448)** |
| **Mean Age at Diagnosis, in *years*** | 74.3 | 74.3 | 73.9 | 70.8 | 73.6 | 73.7 |
| **All-Cause deaths, *n*** | 47 | 59 | 35 | 21 | 62 | 224 |
| **Type of Cholesterol Lowering Drug Use** | | | | | | |
| Never | 61% | 48% | 55% | 44% | 64% | 793 |
| Past | 11% | 6% | 11% | 7% | 4% | 97 |
| Current | 28% | 47% | 34% | 49% | 33% | 558 |
| ***Body Mass Index (*kg/m^2^*)** | | | | | | |
| Normal (18.5-24.9) | 21% | 56% | 22% | 22% | 38% | 542 |
| Underweight (<18.5) | 1% | 2% | 0% | 0% | 3% | 23 |
| Overweight (25-29.9) | 30% | 27% | 39% | 33% | 33% | 453 |
| Obese I (30-34.9) | 38% | 11% | 29% | 36% | 19% | 321 |
| Obese II/III (>35) | 7% | 1% | 6% | 7% | 4% | 61 |
| **Cardiovascular Disease** | | | | | | |
| None | 18% | 30% | 30% | 28% | 36% | 434 |
| Hypertension, or taking hypertension medications | 66% | 59% | 54% | 58% | 53% | 830 |
| History of cardiovascular disease or stroke | 17% | 11% | 16% | 14% | 11% | 184 |
| **Diabetes** | | | | | | |
| No | 80% | 84% | 71% | 76% | 90% | 1191 |
| Yes | 20% | 16% | 29% | 24% | 10% | 257 |
| ***Stage at Diagnosis** | | | | | | |
| Localized | 71% | 80% | 70% | 68% | 71% | 1066 |
| Regional | 23% | 17% | 25% | 28% | 24% | 321 |
| Distant | 6% | 2% | 3% | 3% | 3% | 42 |
| ***ER/PR** | | | | | | |
| ER+/PR+ | 62% | 69% | 65% | 78% | 73% | 1008 |
| ER+/PR- | 11% | 15% | 9% | 11% | 13% | 181 |
| ER-/PR+ | 2% | 1% | 1% | 1% | 0% | 11 |
| ER-/PR- | 22% | 13% | 19% | 7% | 11% | 200 |
| * Percentages may not add up to 100% due to missing values.  ER=Estrogen Receptor, PR=Progesterone Receptor | | | | | | |

| **Supplementary Table 4: Association of cholesterol lowering drug use with all-cause mortality and breast cancer-specific mortality among women diagnosed with breast cancer in the MEC (2003-2014) using inverse propensity treatment weighting^*^** | | | | | |
| --- | --- | --- | --- | --- | --- |
| **Cholesterol lowering drug use** | **n cases** | **n deaths** | **HR** | **95% CI** | **p-value** |
| **All-cause mortality** | | | | | |
| Never (Reference) | 793 | 123 | 1.00 |  |  |
| Ever | 655 | 101 | **0.80** | **0.67-0.95** | **0.01** |
| **Breast cancer-specific mortality** | | | | | |
| Never (Reference) | 791 | 52 | 1.00 |  |  |
| Ever | 654 | 35 | 0.88 | 0.66-1.18 | 0.39 |
| * Adjusted for age at breast cancer diagnosis, race/ethnicity, level of education, body mass index, daily caloric intake, Alternate Healthy Index Score, age at menarche, cardiovascular disease, diabetes, tumor stage, lymph node status, hormone receptor status, surgery, and hormone therapy.  Bolded values indicate p-values ≤ 0.05 | | | | | |
